# Structure-Function Relationships of C-Reactive Protein in Bacterial Infection

**DOI:** 10.3389/fimmu.2019.00166

**Published:** 2019-02-26

**Authors:** Donald N. Ngwa, Alok Agrawal

**Affiliations:** Department of Biomedical Sciences, James H. Quillen College of Medicine, East Tennessee State University, Johnson City, TN, United States

**Keywords:** C-reactive protein, factor H, phosphocholine, pneumococcal C-polysaccharide, *Streptococcus pneumoniae*

## Abstract

One host defense function of C-reactive protein (CRP) is to protect against *Streptococcus pneumoniae* infection as shown by experiments employing murine models of pneumococcal infection. The protective effect of CRP is due to reduction in bacteremia. There is a distinct relationship between the structure of CRP and its anti-pneumococcal function. CRP is functional in both native and non-native pentameric structural conformations. In the native conformation, CRP binds to pneumococci through the phosphocholine molecules present on the C-polysaccharide of the pneumococcus and the anti-pneumococcal function probably involves the known ability of ligand-complexed CRP to activate the complement system. In the native structure-function relationship, CRP is protective only when given to mice within a few hours of the administration of pneumococci. The non-native pentameric conformation of CRP is created when CRP is exposed to conditions mimicking inflammatory microenvironments, such as acidic pH and redox conditions. In the non-native conformation, CRP binds to immobilized complement inhibitor factor H in addition to being able to bind to phosphocholine. Recent data using CRP mutants suggest that the factor H-binding function of non-native CRP is beneficial: in the non-native structure-function relationship, CRP can be given to mice any time after the administration of pneumococci irrespective of whether the pneumococci became complement-resistant or not. In conclusion, while native CRP is protective only against early stage infection, non-native CRP is protective against both early stage and late stage infections. Because non-native CRP displays phosphocholine-independent anti-pneumococcal activity, it is quite possible that CRP functions as a general anti-bacterial molecule.

## Introduction

C-reactive protein (CRP) is a multifunctional molecule of the innate immune system in humans (1–4). CRP is a cyclic pentameric protein comprised of five identical non-covalently attached subunits. Each subunit has an intra-disulfide bond and the molecular weight of each subunit is ~23 kDa (5, 6). A phosphocholine (PCh)-binding site is located on the same face of each subunit in the homopentamer. The amino acids Phe^66^, Thr^76^, and Glu^81^ in CRP are critical for the formation of the PCh-binding site (7–9). Once CRP is complexed with a substance with exposed PCh group, the complex activates the complement system through the classical pathway (10–12).

*Streptococcus pneumoniae* are gram positive bacteria that asymptomatically colonize the upper respiratory tract (1, 13–15). It is the most common bacterium that causes community-acquired pneumonia and is also a significant cause of septicemia and meningitis (1, 13–15). Systemic pneumococcal infection raises the level of CRP in serum by up to several hundred-fold in humans as a part of the acute phase response (16–18). CRP binds to pneumococci through Ca^2+^-dependent interaction with PCh residues present on the pneumococcal cell wall C-polysaccharide (PnC) (19, 20). In mice, however, CRP is only a minor acute phase protein; therefore, mice have been useful in investigating the functions of human CRP *in vivo* (21).

In murine models of pneumococcal infection, passively administered human CRP has been shown to be protective against lethal pneumococcal infection, that is, CRP decreases bacteremia and enhances survival of infected mice (1, 22–26). CRP-deficient mice are more susceptible to pneumococcal infection than are wild type mice, which indicates that the trace level of endogenous mouse CRP is capable of exerting anti-pneumococcal functions (27). Mice transgenic for human CRP are also protected against infection with *S. pneumoniae* (28). The mechanism of anti-pneumococcal action of CRP in mice, however, is unknown.

Current research on defining the mechanism of anti-pneumococcal actions of CRP benefited from a key finding made several decades ago using passive administration of purified human CRP into mice (29). CRP was protective when injected into mice 6 h before to 2 h after the administration of pneumococci. CRP was not protective when mice received CRP 24 h after infection, suggesting that CRP is protective during early stage infection but not in late stage infection. For early stage protection, it is believed that the mechanism of action of CRP involves the capability of CRP to bind to pneumococci through PCh groups present on their surfaces and subsequent activation of the classical complement pathway by pathogen-bound CRP. Obviously, this mechanism does not operate for late stage infection. A PCh-independent mechanism for anti-pneumococcal function of CRP has been proposed along with an explanation for the inability of CRP to be protective against late stage infection (1, 24–26). In this article, we review PCh-dependent, PCh-independent, and other proposed mechanisms for the anti-pneumococcal function of CRP during both early stage infection (when CRP and pneumococci are administered into mice 30 min apart) and late stage infection (when CRP and pneumococci are administered into mice 24 h apart).

## PCh-Dependent Anti-Pneumococcal Function of CRP

*In vivo* experiments employing a CRP mutant incapable of binding to PCh, PnC, and whole pneumococci provided results indicating that CRP-mediated protection of mice against infection is independent of binding of CRP to PCh; the CRP mutant was as effective as wild-type CRP in protecting mice against early stage infection (26). The PCh-binding mechanism, however, does contribute to the protection of mice during the early stage of infection (25, 26). The PCh-dependent mechanism contributes to the initial and immediate clearance of pneumococci as has been shown employing a variety of murine models of infection (26, 27). Overall, the combined data suggest that both PCh-dependent and PCh-independent mechanisms operate in the protection of mice against early stages of infection, although the PCh-dependent mechanism is not necessary (25, 26).

Indirect evidence has been presented to show the importance of the PCh-binding property of CRP and subsequent complement activation by CRP-complexes in protection from infection. It has been shown that CRP binds to gram negative bacterial lipopolysaccharide (LPS) if the LPS is modified by adding a few PCh residues to it. The binding of CRP to PCh-modified LPS increases based on the number of PCh residues added and subsequently affects the resistance of the organism to the killing effects of serum (30). Also, the pneumococcal surface protein PspA, which is a choline-binding protein, is known to bind to PCh. PspA thus competes and inhibits the binding of CRP to PCh on pneumococci and decreases complement activation (31). Similarly, pneumococci growing as a biofilm are avirulent due to a decrease in PnC production although with an increase in PCh expression, interference from pneumococcal surface protein PspC, reduced binding of C1q to CRP-PCh complexes, and subsequent failure to activate complement (32, 33). Biofilm formation in *S. pneumoniae* is an effective means of evading complement attack (33).

One study suggested that the property of CRP to activate the classical pathway of complement in human serum is irrelevant for the protective function of CRP in mice infected with *S. pneumoniae*, because human CRP does not activate murine complement via the classical pathway (23). Since complement-deficient mice do not show CRP-mediated protection to pneumococcal infection (34), it is possible that CRP-complexes are able to activate murine complement system via a pathway other than the classical pathway (1, 23). It has been proposed that human CRP-complexes are able to activate the lectin pathway in murine serum and are able to activate both the classical and lectin pathways in human serum (23). CRP has been shown to interact with both L-ficolin and M-ficolin and activate the lectin pathway of complement (35–39). The interaction between CRP and L-ficolin increases 100-fold under the conditions of slight acidosis and reduced calcium levels, and it has also been shown that the cross-talk between CRP and L-ficolin mediates killing of *Pseudomonas aeruginosa* in plasma (37). L-ficolin also recognizes PCh on pneumococcal strains and triggers activation of the lectin complement pathway (40). Lectin-like oxidized LDL receptor, LOX-1, can also recognize CRP and is involved in CRP-dependent complement activation (41, 42). CRP is a major hemolymph protein in the horseshoe crab *Carcinoscorpius rotundicauda*. When CRP is in the hemolymph, it binds to a range of bacteria through galactose-binding protein and ficolin. Accordingly, it has been proposed that CRP does not act alone but collaborates with other plasma lectins to form stable pathogen recognition complexes when targeting a wide range of bacteria for destruction (35).

## PCh-Independent Anti-Pneumococcal Function of CRP

Factor H, a regulator of complement activation, has been implicated in resistance of pneumococci to complement attack (43, 44). Factor H protects from complement attack by inhibiting the activation of the alternative pathway on host cells and on those pathogenic surfaces which are capable of recruiting factor H from the plasma. On the host cells, factor H binds to polyanionic structures and glycoproteins found on the cell surface (45). On *S. pneumoniae*, factor H binds to a surface protein called Hic (factor H-binding inhibitor of complement) which is a variant of PspC (46, 47). Thus, pneumococci use factor H to evade complement-mediated killing. The recruitment of factor H by pneumococci might be the reason why CRP does not protect mice from pneumococcal infections during late stage infection.

CRP does not bind to factor H under normal physiological conditions (48–52). Denaturation conditions for CRP enable CRP to bind to factor H (4, 48–51). For example, immobilization of CRP on to a surface enables CRP to bind to factor H (4, 53, 54). Monomeric CRP (mCRP) also binds to factor H, in a Ca^2+^-independent manner (55). The Y384H polymorphism of factor H affects binding affinity for mCRP. CRP binds to factor H-Tyr^384^ more strongly compared to factor H-His^384^ which is the risk allele (56–60). PCh does not compete with factor H for binding to CRP (52). It has been suggested that when CRP immobilizes itself on *S. pneumoniae*, it limits excessive complement activation by recruiting factor H (61, 62). CRP has also been shown to modulate lectin pathway-dependent cytolysis by recruiting factor H (63, 64). When CRP binds to dead cells it does not recruit factor H (55). mCRP also binds to factor H-related proteins (FHR) FHR1 and FHR5 and to factor H like protein 1 (FHL-1) which inhibit subsequent recruitment of factor H (65–68). CRP has also been shown to recruit factor H on other cell types, for example, CRP recruits factor H after binding to collectin CL-P1 on the surface of placental cells (69, 70). Otherwise, the interaction of CRP with CL-P1 activates the classical complement pathway. The interaction of CL-P1 with factor H might be the key to prevent self-attack due to complement activation induced by the CL-P1 and CRP interaction (69, 70).

Based on results obtained from the experiments performed under defined conditions—native pentameric CRP does not bind to factor H while mCRP binds to factor H—it was hypothesized that a non-native pentameric CRP may also be able to bind to factor H (48). Indeed, the native pentameric structure of CRP could be modified *in vitro* to generate non-native pentameric CRP capable of binding to factor H (2, 48–50). Since non-native CRP and Hic can bind to factor H simultaneously, it is possible that non-native CRP can bind to factor H-coated pneumococci, cover the factor H-Hic complex formed on bacteria and therefore eliminate the repressive effect of factor H on complement activation (71–73). Recently, a CRP mutant capable of binding to immobilized factor H was evaluated for its ability to protect against late stage pneumococcal infection. The CRP mutant protected mice against infection regardless of the time of administration into mice (71–73). These data lead to the proposal that the PCh-independent mechanism first involves a structural change in CRP which is then followed by the interaction between structurally altered CRP and factor H-bound pneumococci. Once factor H on pneumococci is bound to structurally altered CRP, such pneumococci may not be resistant to complement attack any longer (1, 71–73).

Besides, factor H, *S. pneumoniae* have also been shown to recruit another complement inhibitor, C4b-binding protein (C4BP) via Hic that also recruits factor H (74, 75). Pneumococci also use another cell surface protein, enolase, to recruit C4BP (75). By recruiting C4BP, pneumococci are able to evade complement attack. We hypothesize that non-native CRP may also be protective against those pathogens which recruit C4BP for complement evasion: non-native CRP could bind to factor H/C4BP-coated pneumococci, and then the complex formed by CRP, factor H/C4BP, and Hic could activate the lectin pathway of complement and trigger killing of the pneumococci. The possibility cannot be ruled out that the PCh-independent mechanism may involve the binding of non-native CRP to pneumococcal surface proteins, as CRP has been shown to interact with several choline-binding proteins found on pneumococci in a Ca^2+^-independent manner (76).

## CRP as an Anti-Bacterial Molecule

CRP binds to several pathogenic serotypes of *S. pneumoniae* (77–79) and binds more avidly to those strains which contain PCh in both cell wall and capsular polysaccharides, such as type 27 (80). CRP, like lectins, also reacts with polysaccharides that do not contain PCh, such as depyruvylated type-IV capsular polysaccharide prepared from type 27, in the presence of calcium, and probably the reaction is due to N-acetylgalactosamine in the polysaccharide (81–84). CRP appears to have opsonin properties; it causes agglutination and lysis of gram positive bacteria *Staphylococcus aureus, Bacillus subtilis, Streptococcus pyogenes*, and *Streptococcus agalactiae* (77, 78).

The anti-bacterial action of CRP is not limited to gram positive bacteria only. CRP also protects mice from the early stages of infection with *Salmonella enterica* serovar Typhimurium, which is a gram negative bacterium and to which CRP does not bind *in vitro* (85). But CRP has been shown to bind to *S. enterica* in the presence of serum (35). CRP also binds to *Haemophilus influenzae* (86). *H. influenzae* undergoes phase variation in expression of the PCh on the cell surface-exposed outer core of the LPS. PCh-positive variants are more sensitive to the bactericidal activity of human serum which requires the binding of serum CRP to whole bacteria with subsequent activation of complement (86–88). The ability of *H. influenzae* to vary PCh expression to zero may relate to its ability to cause invasive infection by evading attack by CRP (86). Mouse models of *H. influenzae* infection have not been established yet to determine whether CRP protects against infection with *H. influenzae* (27). CRP also binds to *Neisseriae spp*. in a Ca^2+^-dependent manner (89–91). PCh is present on the LPS of several species of commensal *Neisseriae* and, like *H. influenzae, Neisseriae* also undergo phase variation in expression of the PCh on their LPS (91). Mouse protection experiments have not been performed for *Neisseriae* either, employing native or non-native pentameric CRP.

Some experiments suggest a role of CRP in protecting animals against lethal toxicity of LPS, although the subject has been controversial (92–96). In the hemolymph of horseshoe crab, *Carcinoscorpius rotundicauda*, CRP was identified as the major LPS-binding protein in infections with *Pseudomonas aeruginosa* (97). CRP bound to all bacteria tested in the horseshoe crab hemolymph (35). The binding of CRP to LPS is indirect; a third molecule called galactose-binding protein (GBP) participates in bridging CRP and LPS (98). Upon binding to LPS, GBP interacts with CRP to form a pathogen-recognition complex, which helps to eliminate invading microbes (35, 98). Combined data raise the possibility that CRP functions as a general anti-bacterial molecule; CRP may require a change in its pentameric conformation and also seek help from other serum proteins to form pathogen-recognition complexes.

## CRP as an Anti-Inflammatory Molecule

Native pentameric CRP can dissociate into mCRP via an intermediate non-native pentameric structure (50, 99–101). All three forms, native pentameric, non-native pentameric, and mCRP display different ligand recognition functions *in vitro* (2, 102–104). Under conditions of low pH, reduced calcium levels and oxidation-reduction, CRP is converted to a non-native conformation but remains pentameric (48–50, 105–107). When non-native CRP binds to a non-PCh ligand, it denatures further to mCRP. Similarly, when CRP binds to cell membranes, liposomes, and cell-derived microvesicles, it undergoes a structural change which involves spatial separation of the monomers from each other without disrupting the pentameric symmetry to form a transitional state CRP (108). The mechanism by which CRP recognizes membrane lipids and binds in a Ca^2+^-independent manner depends on the combination of protein form, lipid composition, and membrane shape (109, 110). Surface-immobilization of CRP generates a preservable intermediate with dual antigenicity expression of both CRP and mCRP. The intermediate exhibits modified bioactivities, such as a high affinity with solution-phase proteins (107). It has been shown that mCRP but not CRP is the major isoform present in local inflammatory lesions (111). Since mCRP is insoluble, it is considered a tissue-bound form of CRP. Thus, an intermediate stage of CRP structure seems to be responsible for anti-inflammatory host defense functions of CRP *in vivo*. Structural changes *in vivo* may be converting CRP into an anti-inflammatory molecule assuming that the ultimate pro-inflammatory by-product, mCRP, is continuously being removed. An intrinsically disordered region of amino acid residues 35–47 in CRP is responsible for mediating the interactions of mCRP with diverse ligands (112), and possibly also responsible for mediating the interactions of non-native pentameric CRP with diverse ligands (48–50).

## Conclusions

While native CRP is protective only against early stage infection, non-native pentameric CRP is protective against both early stage and late stage infections in murine models of pneumococcal infection. Because non-native pentameric CRP displays PCh-independent anti-pneumococcal activity, it is quite possible that CRP functions as a general anti-bacterial molecule. Thus, pentameric CRP is an anti-inflammatory molecule.

A long-term goal could be to focus on the discovery and design of small-molecule compounds to target CRP, a compound that can change the structure of endogenous CRP so that the structurally altered CRP is capable of binding to factor H-bound pneumococci. A recent study showed that injections of sub-inhibitory concentrations of antibiotics enhanced the binding of CRP to three antibiotic-resistant *S. pneumoniae* strains in serum and enhanced antibody-dependent complement activation (113). Based on these findings, another goal could be to investigate the effects of combinations of non-native pentameric CRP with various antibiotics in pre-clinical studies.

## Author Contributions

All authors listed have made a substantial, direct and intellectual contribution to the work, and approved it for publication.

### Conflict of Interest Statement

The authors declare that the research was conducted in the absence of any commercial or financial relationships that could be construed as a potential conflict of interest.

## Abbreviations

CRP: C-reactive protein
FHR: factor H-related protein
LPS: lipopolysaccharide
mCRP: monomeric CRP
PCh: phosphocholine
PnC: pneumococcal C-polysaccharide.
